# Revisiting the Village Where Arsenic Contamination of Underground Water Was First Discovered in Bangladesh: Twenty-Five Years Later

**DOI:** 10.3390/ijerph18010259

**Published:** 2020-12-31

**Authors:** Maiko Sakamoto

**Affiliations:** Department of International Studies, Graduate School of Frontier Sciences, The University of Tokyo, Chiba 2778563, Japan; m-sakamoto@k.u-tokyo.ac.jp

**Keywords:** arsenic contamination, underground water, risk perception, risk communication, questionnaire survey, community-level analysis, logistic regression, social capital, mitigation policy

## Abstract

A survey was conducted in an As-affected village of Bangladesh—the first discovery of As contamination in the country—to assess the current situation and how implementation activities have worked to mitigate the problem. The As testing showed that the levels were less than the Bangladesh standard (50 µg/L) in all shallow tube-wells throughout the village. The questionnaire survey was conducted in the village as well as a neighboring As-affected village for comparison. The results revealed that there was a significant number of people using shallow tube-wells in both villages despite knowing that these wells could be contaminated with As and that safe water was available through a pipeline water supply. About 70% of responding households possessed their own water sources, mostly shallow tube-wells, and owners were less likely to choose tap water for drinking purpose than nonowners. In the village where As contamination was first reported, those individuals with a higher level of education and strong ties with neighbors were more likely to use shallow tube-well water for drinking purposes rather than tap water. This study suggests several measures to mobilize people to get safe water, namely providing subsides to install private taps, supplying public taps, and marketing and distributing handy water quality tests for households.

## 1. Introduction

Arsenic contamination in underground water has been a serious health risk for those populations in many developing countries who have relied on underground (well) water due to an insufficient safe drinking water supply infrastructure. Among South Asian countries, identification of As contamination in groundwater and the effect of its continuous intake on health was reported in 1983 when a patient in West Bengal, India, was diagnosed with arsenicosis [1]. It was presumed that Bangladesh was also the victim of As contamination, being in close proximity to West Bengal and having similar geological and hydrological conditions. Subsequently, As contamination of tube-well water in Bangladesh was first confirmed in 1993 in Baroghoria Union, Chapai Nawabganj District by the Department of Public Health Engineering (DPHE) of the Government of Bangladesh, and eight arsenicosis patients were reported in 1994 [2]. Since then, many international aid organizations have supported the Bangladeshi government in the mitigation of this problem in collaboration with domestic institutions and organizations. A variety of mitigation activities have been implemented such as the installation of alternative water sources (such as deep tube-wells, dug wells, pond sand filters, As removal plants, and pipelines), as well as information dissemination on the harm of As. The mitigation activities also included As testing of tube-wells in the 64 districts of Bangladesh, which identified As presence in drinking water in 61 of 64 districts in Bangladesh [3]. Tube-wells were painted green if the level of As contamination was below the Bangladesh standard threshold (50 µg/L) or red if it was above the threshold [4]. Hereafter, water containing less As than the threshold level is called “safe,” whereas water with more than the threshold level is called “contaminated.”

According to the DPHE’s annual report [5], 87% of the population are secured with community water sources, which include shallow tube-wells, deep tube-wells, pond sand filters, and dug wells. Pipeline water supply is now an option, and the promotion of its construction in As-affected areas was stated in the 2014 National Strategy for Water Supply and Sanitation, although pipeline water supply as a standard component of infrastructure in rural areas of Bangladesh is still a long way off [6]. Despite the efforts made so far, reports have shown that one-third of these As-safe water options are not in use or have been abandoned [7,8,9]. The World Bank has conducted water and sanitation projects in Bangladesh since 1998, and in the report on the project contribution from 2012 to 2017, it stated that “While improved water supply coverage in rural Bangladesh is now above 97 percent, water quality still poses a significant challenge. Thirteen percent of the country’s water sources have As levels above the threshold that the government defines as dangerous” [10]. Sustainability of groundwater source quality is another concern. Although a study suggested that the As levels in tube-well water were fairly stable over time and that deep tube-wells were less likely to be contaminated with As [11], several recent studies have suggested the possibility of As invasion into deep groundwater aquifers due to unrestricted long-term pumping [12,13,14]. As such, the As contamination of groundwater remains a serious problem to be tackled by the country.

The present study aims to explore how the implementation actions have worked to mitigate the problem by looking into the village where As was first discovered in Bangladesh, Chamagram Village, in Baroghoria Union, Chapai Nawabganj District. It is worth revisiting the first reported site to determine the existing gaps because the village has been exposed to many interventions after the initial discovery of As 25 years ago. Tests on tube-well water were conducted in 2018 in the village to determine the level of As contamination. Concurrently, a questionnaire survey was conducted in the village, as well as in the neighboring village known for As contamination, to better understand local contexts. Section 2 provides a summary of the past As test results implemented in the study area as well as background information on the surveyed villages. Section 3 explains the methodologies used, including the survey design and As testing. Section 4 reports the results of the As tests, questionnaire survey, and logistic regression analysis on villagers’ water source selection behavior, which are discussed in Section 5. Section 6 provides a summary and conclusion.

## 2. Background Information on Study Sites

**Figure 1:**
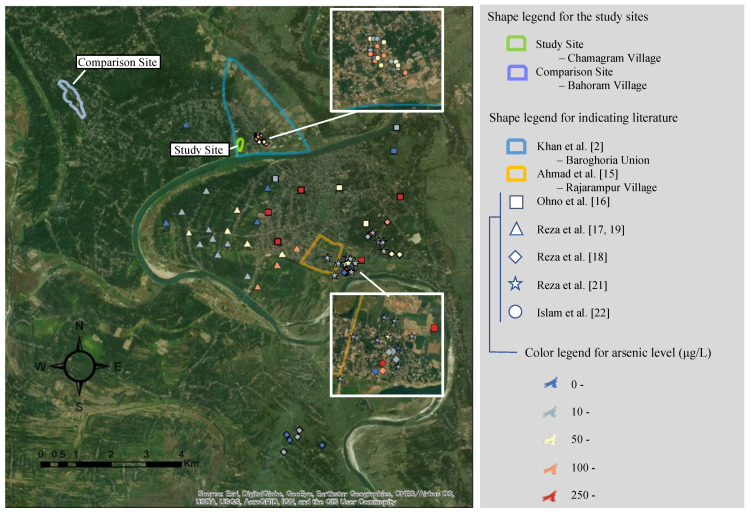
Map of tested tube-well locations and field-testing sites.

**Figure 2:**
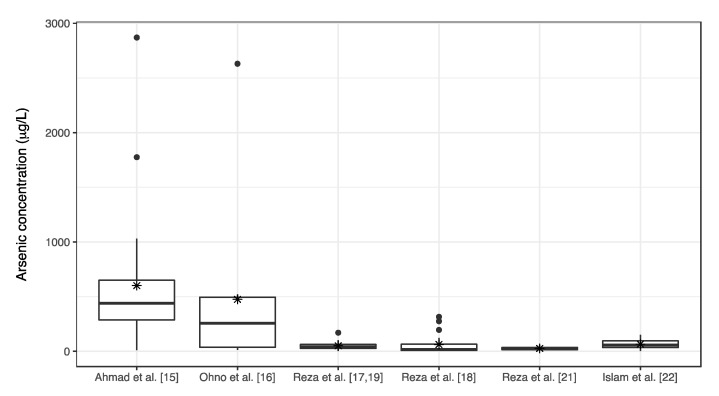
Boxplots of As concentrations from previous scientific literature data. The boxplots show the “minimum”, first quartile (Q1), median, third quartile (Q3), and “maximum” values where the maximum value is determined by Q3 + 1.5 × (Q3 − Q1) and the minimum values is determined by Q1 − 1.5 × (Q3 − Q1). Black dots and asterisks represent outliers and mean values, respectively.

Several previous studies have reported the level of As in underground water in the studied region. Figure 1 shows a remote sensing image of the area, in which the blue line indicates Baroghoria Union where Khan et al. [2] reported the As patients in 1994. The locations and the As levels of the tube-wells tested in other studies are also shown. The case study reported by Ahmad et al. [15] provided the As concentrations for tube-wells in the district but, as the specific locations of the tested tube-wells were not given, only the area within which the studied villages are located is shown in Figure 1 by the orange line. Table 1 summarizes the results of the As tests reported in the studies and Figure 2 shows the boxplots of the results. As shown in Table 1 and Figure 2, the concentration of As in the area decreased after the early 2000s.

Figure 1 also shows the locations of the sites in the present study. The study site is located in Chamagram Village where As was first detected in Bangladesh in 1993. The survey was conducted in the known hotspot area of As contamination of the village where the pipeline water supply was provided. Another village was selected for the survey to better understand local contexts and people’s behaviors and risk perceptions related to As contamination. The other survey site is located in Bahoram Village, Ranihati Union, Chapai Nawabganj District. The site was selected because it is known for its As contamination and for being served with pipe water. Hereafter, the survey site in the village where As was first reported is called the study site and the survey site in the other village is called the comparison site. In the study site, the water supply service was installed and has been run by a domestic NGO, Brotee, since 2009 and is assisted by the Social Development Foundation, a government foundation under the Ministry of Finance. It has operated under the Public-Private Partnership arrangement. The average daily demand is estimated as 135,000 L/day and production capacity of the water treatment plant is 300,000 L/day. The water source is surface water drawn from the Mahananda River. The raw water is purified with the up-flow roughing filter and the slow sand filter. The water quality is tested for fecal coliform, total coliform, turbidity, and PH on an occasional basis (not as a routine). The treated water is provided through a pipeline network of 6.6 km in length every day from 3:30 p.m. to 8 p.m. The monthly tariff is Bangladeshi Takas (BDT) 320 per household. Along with the pipeline water supply, dug wells were given by the government as the intervention. In the comparison site, the pipeline system was constructed by the Rural Development Academy (RDA), Bogra, in collaboration with the Institute of Water Modeling (IWM) in 2013. After the construction, it was handed over to the local government, and they have operated and managed the water supply system since then. The existing overhead tank capacity is 30,000 L, but to satisfy the daily demand of 90,000 L, the water pumps are operated three times a day. Underground water from the shallow aquifer is used. The water is distributed without any treatment. The domestic NGO, DASCOH, regularly checks the water quality, particularly for As. So far, As contamination of the source water has not been reported. The pipeline network is 15 km in length and the water is provided through the network three times per day for about 1.5 h each time. The monthly tariff is BDT 100 per household. Along with the pipeline water supply, dug wells were provided by the government, and As-removal filters (called SONO filters) were provided by DASCOH as the interventions. In both villages, taps are supplied for individual properties and there are no public taps. Thus, the villagers have to the installation cost of the taps. The minimum installation cost is about BDT 7000 BDT and the maximum is BDT 35,000. The cost mainly varies depending on the size of the water storage tank that villagers often install on rooftops. A subsidy is not provided, so the villagers have to bear all the relevant costs by themselves.

In the study site, 49 out of the 74 shallow tube-well owners answered that they had experienced the As testing on their own shallow tube-wells, implemented either by the local government or NGOs about 6–20 years ago, and only 5 were found to be As-contaminated. In the comparison site, 70 out of the 92 shallow tube-wells owners answered that their tube-wells were tested once and 47 were found to be contaminated. The information about As-contaminated patients further confirmed the situation: The respondents in the study site answered that no one developed arsenicosis symptoms among family members or among neighborhood villagers, whereas in the comparison site, 45 out of the 154 respondents answered there were As-contaminated patients either in their family or in the neighborhood.

## 3. Materials and Methods

The preliminary survey was conducted in July 2017 to understand local situations and select a comparison site. The questionnaire survey was conducted in February 2018. Questions were asked to the wives of the householders. The women were selected as respondents because they were considered responsible for choosing the drinking water source for their family’s use. The survey included questions about the family profile, occupations, financial status, water source options, and perceptions of risk regarding As in the local water supply. The survey did not intend an intervention, so the ethical approval was not obtained in advance. The verbal informed consent was obtained due to the high rate of illiteracy. Logistic regression analysis was applied to the collected data to investigate villagers’ perceptions and behaviors related to the As risk and selection of drinking water source. R version 3.3.1 with the function “glm” was used for logistic regression, where the error structure assumed was “binominal distribution” and the link function was set as “logit” (link = logit) [23].

The As tests were conducted for all the tube-wells in the study site with the HACH EZ Arsenic Kit. For confirmation of the results, 10 randomly selected samples from the study site and samples from 17 tube-wells installed in the adjacent village and previously tested by Islam et al. [22] were sent to the DPHE laboratory for more accurate testing. The location of the previously tested wells in the adjacent village is shown in Figure 1 (not a comparison site). An effort was made to identify the 18 tube-wells tested in the previous study based on the location information provided in the published paper. Of these, 13 were identified, 4 were not located, and 1 had been demolished. Therefore, the 13 identified tube-wells that had been tested in the previous study and 4 additional tube-wells situated near the previously tested (but unlocated) tube-wells were tested in the DPHE laboratory. Water samples for the lab test were stored in polypropylene bottles and preserved by acidifying with concentrated hydrochloric acid maintaining pH level < 2. The As concentration was analyzed by Hydride Vapor Generating (HVG) Atomic Absorption Spectrometry (SpectrAA-220, Varian). The As field test was conducted concurrently with the questionnaire survey in February 2018, and the DPHE lab tests were conducted in March 2018 for the 10 samples from the study site and in April 2018 for the 17 samples from the previously studied village.

## 4. Results

### 4.1. As Levels in Underground Water

The results of the HACH EZ Arsenic Kit tests in the study site showed that all the shallow tube-well water was found to be light yellow or almost colorless, which, according to the color chart indicating the concentration of As, suggested all the tube-wells contained less As than the Bangladesh standard threshold (50 µg/L). The results of the DPHE laboratory testing, as well as the coordinates of each of the tested wells, are shown in Table 2. This testing confirmed that the result of the field test kit was fair and none of the tube-wells contained more As than the permissible threshold for drinking water. Table 2 also shows the result from and the coordinates of each tested well in the previous study site. The results suggest that all the tube-wells contained quite low levels of As.

### 4.2. Socioeconomic Status and Risk Perceptions

In the study site, the responses to the questionnaire were collected from almost all the households who were present at the time of the survey participated in the survey. In the comparison site, which is a part of a large village, the survey was conducted by the cluster-wise method until the sample size reached the sufficient number needed for statistical analysis. Almost all of the households in every cluster participated.

The descriptive statistics of the questionnaire survey results are summarized in Table 3. As shown in Table 3, about 37% of households used tap water for drinking purposes and 62% used nonfiltered shallow tube-well water in the study site. In the comparison site, about 60% used tap water and 38% used nonfiltered shallow tube-well water.

**Figure 3:**
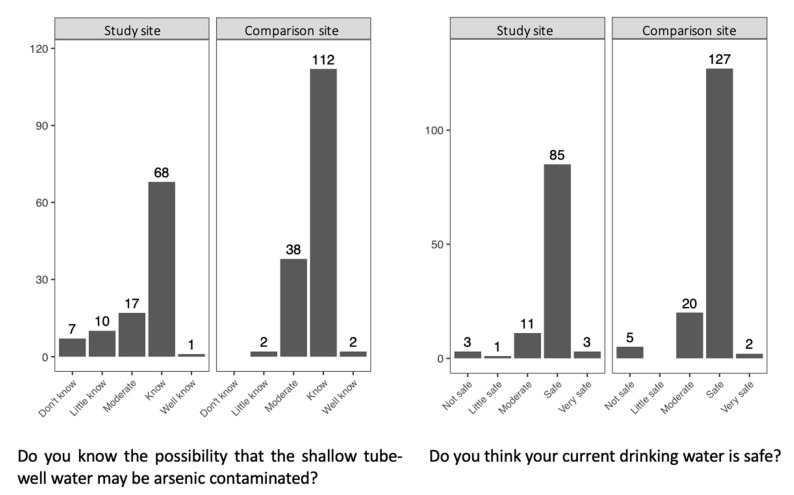
Comparison of villagers’ perceptions of As-related risk. The answers in the left figure show the degree of knowledge. *Little know* was recorded when a respondent had information but did not seem to understand it and hesitated to answer *Don’t know*. *Moderate* was recorded when a respondent had information and understood it but did not seem to believe it and made confusing statements. *Know* was recorded when a respondent had information and understood it. *Well know* was recorded when a respondent had information and was able to explained it well.

Figure 3 shows the villagers’ risk perceptions. Concerning the possibility that a shallow tube-well might be contaminated with As, some households (16.5%) in the study site and almost none (1.3%) in the comparison site reported that they were not aware of the potential contamination. Furthermore, although many people (71.2%) knew of the possibility that shallow tube-wells might be contaminated with As (the left figure of Figure 3), many of the respondents (63.1%) from the study site drank shallow tube-well water nonetheless (Table 3) and answered that their drinking water was safe (the right figure of Figure 3).

Table 4 shows the relationships between the water source uses and possession status. As for the drinking water source choices, when respondents possessed only taps, they were more likely to use their own water source (Tap). However, possessing only shallow tube-wells or both of taps and shallow tube-wells, the revealed tendency was different between the two villages. The respondents of the study site were more likely to choose their own water source (shallow tube-wells) than those of the comparison site. The difference in the water source use for the shallow tube-well owners between the two villages was examined by the chi-square test for the cross tabular and was found statistically significant with *p* < 0.01. The choice of the nonowners was similar in the two villages: They were more likely to choose tap water. Interestingly, the owners from the study site tended to use different water sources for drinking, cooking, and bathing purposes. However, nonowners from the study site, as well as owners and nonowners from the comparison site, tended to use the same water sources for the different purposes. The dominant reason of using shallow tube-wells instead of taps was “Because it is expensive” (80.8%) and the second dominant reason was “I don’t like the taste” (12%).

Considering the previous As testing results and the nonexistence of As patients in the study site, it is natural that the respondents from the study site tended to use shallow tube-wells rather than taps. However, the villagers usually had knowledge about the possibility of As contamination of shallow tube-wells in the area. Therefore, considering the conditions of the study site, including the many interventions (such as information dissemination) and the fact that the villagers likely had higher levels of education and income than those from the comparison site, it is also natural to expect that those from the study site had a better understanding of the risks of shallow tube-well water and took more risk-averse behaviors than those from the comparison site.

### 4.3. Logistic Regression Analysis on Tap Water Installation and Water Source Selection

To further investigate villagers’ perceptions and behaviors against the As risk, logistic regression analysis was conducted on the tap water installation, as well as their water source selection for drinking and cooking purposes, where the significantly different choice behaviors were observed in the study site. For the regression analysis on tap water installation, the dummy variable of tap installation status (0 = not possessing a tap, 1 = possessing a tap) was used as the dependent variable, whereas for the regression analyses on water source selection, the dummy variable of water source (0 = shallow tube-well water, 1 = tap water) selected by respondents for drinking and cooking purposes was used. Because the majority of the respondents were aware of the risks of shallow tube-wells in the region, as Figure 3 suggests, these dummy variables can be regarded as a proxy of a risk attitude. As for the explanatory variables, six variables were included: Log of income, Education, Feeling physical burden, Knowledge on shallow tube-well, and Social capital. These were often considered as relevant variables to risk perceptions and behaviors. In the following results, the variance inflation factors (VIF) for all the explanatory variables in each model were calculated, and it was confirmed that multicollinearity happens less likely between the variables.

We used the partition approach [18] for the inclusion of the applied interaction terms where the products between the interested explanatory variables (Education, Knowledge, and Social capital) and the dummy variables (Village and Possession) with mutually exclusive and exhaustive categories are included. In the most often used method (called the base approach [24]), which includes the main effect along with the interaction terms, the way of interpreting the coefficients for the main effect and interaction terms is not straightforward, particularly for the cases with higher-level interaction terms. In the partition approach, we can interpret the meaning of the coefficients on the interaction terms simply as the effects of the interested explanatory variables on the dependent variable for each profile represented by the combination of dummy variables.

Table 5 shows the logistic regression results on tap water installation. The four models were tested with different specifications. In models (2–4), the interaction terms with the variable “Village” were included to see if the effects are significantly different between the two villages. Overall, the results suggest that none of the explanatory variables were associated with the tap water installation. The low McFadden’s pseudo r-squared shows that the regression models did not fit the data well. This could be because the number of the tap owners was small and important variables for predicting tap water installation behaviors were missed, including access to authorities (local government or nongovernmental organizations (NGOs)), geographical conditions, etc.

Table 6 and Table 7 show the results on the five logistic regression models for each water use purpose with different specifications.

As for the drinking water source selection, models (1) and (2) are the baseline models that include the dummy variables “Village (0 = Study site, 1 = Comparison site)” and “Possession (0 = No, 1 = Yes),” respectively. The variable Possession represents the status of the water sources possession. The result suggests that income, education level, knowledge on the possibility of As contamination of shallow tube-wells, and social capital were not significant for water source selection. However, “Feeling physical burden” was statistically significant, and the sign of the coefficient was negative. The result suggests that people who use shallow tube-wells as a water source, which requires pumping, feel more burdened than those who use taps. This is in line with the result from previous studies [25,26]. The result also suggests that although tube-well users feel burdened in fetching water, they tend to keep using tube-wells without installing taps and do not ask tap-owners for permission to use their taps. Village and Possession were significant in models (1) and (2), respectively. The sign of the coefficient for Possession was estimated as negative. As shown in Table 4, the nonowners’ tendency (more likely to choose taps for drinking purpose) and the owners’ tendency (more likely to choose shallow tube-wells for drinking purpose) contributed to the result. The sign of the coefficient for Village indicates that the respondents from the comparison site were more likely to select tap water than those from the study site. To further investigate the influence of socioenvironmental effects on the water sources selection, models (3)–(5) were developed. In each model, the interaction terms of “Village” and “Possession” were included to test how the influences of education, knowledge, and social capital on water source choices are different between the villages, as well as on the possession status of water sources.

The result of model (3), which includes the interaction between the terms “Village,” “Possession,” and “Education,” suggests that education worked to induce the risk mitigation behavior (i.e., using tap water as the drinking water source) only for nonowners in the comparison site, whereas education worked inversely for owners in the study site and was more likely to induce the risk behavior (i.e., using shallow tube-well water), as the owners had a higher level of education. In model (4), which includes the interaction between the terms “Village,” “Possession,” and “Knowledge,” the result suggests that the knowledge of the possibility of As contamination of shallow tube-wells worked to induce the risk mitigation behavior only for nonowners in the comparison site. This corresponds to the result on the interaction term with “Education.” In model (5), which includes the interaction between the terms “Village,” “Possession,” and “Social capital,” the result shows that social capital had a significant association with water source selection only for owners in the study site, and the association was found to be negative. The result implies that those in the study site who relied more on people in their neighborhood tended to use shallow tube-wells.

As for the analysis result on cooking water source selection shown in Table 7, education, knowledge, and social capital had significantly different effects on the water source choices for the owners in the study site. A large number of owners in the study site tended to use tap water for cooking purposes, although they were more likely to use shallow tube-wells for drinking purposes. Regarding the associations with explanatory variables, education and social capital had no significant effects on cooking water source selection for owners in the study site, whereas they had significant and negative associations on drinking water source selection.

## 5. Discussions

### 5.1. Possible Reasons for the Decreasing Trend in As Levels in the Study Site

The reason why the As level decreased around the study site is in question. First, it has been pointed out that intensive pumping for irrigation is likely to be one of the reasons for As intrusion [27]. The region is famous for mango production, and as we can confirm from the remote sensing images shown in Figure 1, in which the thick green areas are mango forest areas, mango production is widely spread. Khandoker et al. [28] reported that, in their interview of 60 randomly selected farmers from the Chapai Nawabganj district, on average, 55% of the land has shifted from cereal crops to mango cultivation. In addition, we can see that land use in the northeastern part of the union where the study site is located has shifted from open fields to the square brown lots that are brick construction sites. Combined with the answers of people from the study site about their occupations shown in Table 3, we can confirm that there is almost no agricultural land remaining near the study site on which to farm. Massive land use change from agricultural use with irrigation to mango production, which does not require irrigation or nonagricultural use, has taken place in the region. Second, the source of any pipeline-supplied water could be another factor. The pipeline system constructed near the study site uses surface water, whereas the one near the comparison site uses groundwater from the shallow layer, which could change the pressure balance underground and may induce As dissolution into the groundwater. In any case, it is hard to draw any conclusions from the limited information available at present, and further hydrogeological study is necessary to draw conclusions. If the primary cause is either land use or water source selection for the pipeline supply, this implies that some mitigation measures exist that could effectively decrease the As concentration in groundwater.

### 5.2. Determinants of Water Source Selection

Several studies have reported that local people show a strong stated preference for piped water [26,29,30]. However, the results of the present study demonstrated that in both villages, there was a significant number of people using shallow tube-wells despite having access to safe water through the pipeline water supply. The important implications of the logistic regression analysis on the drinking water source selection were as follows: (1) There were no positively associated factors with the tap water source selection in the general analysis; (2) the community-level analysis provided the essential insights for water source selection; and (3) knowledge-induced risk mitigation behaviors for a limited number of population, whereas education level and social capital had a negative effect on risk mitigation behaviors for a certain group of respondents. The previously reported effects of education, knowledge, and economic status on risk mitigation behaviors were split: Positive associations were found for education [31,32], knowledge [32,33], and economic status [32], whereas the effects were rather insignificant for education [33], knowledge [34], and economic status [31,33]. Social capital had a positive association with risk mitigation behaviors [25,32]. In the present study, the effects of these variables were rather insignificant. Still, when we looked at the result from a community-level perspective, different stories were discovered. Simpson [35] pointed out that when groups have different characteristics, the analysis with the aggregate sample may lead to a misleading result (known as Simpson’s paradox). AIC (Akaike Information Criterion) and McFadden’s pseudo r-squared model statistics suggest that the models considering the group differences perform better. The community-level analysis is more suitable to understanding the real dynamics of the problem where socioeconomic and environmental conditions (e.g., topographical and hydrogeological) and intervention history are different between places.

The reason why education level and social capital had a negative effect on the tap water source selection for drinking purposes of owners in the study site is probably related to the information shared among them in advance. In the study site, about 90% of the tested wells among the owned wells were not found seriously contaminated with As in the past, and this, combined with the fact that there had been no arsenicosis cases in the village since some time previously, made them feel comfortable enough to keep drinking the shallow tube-well water and not to use taps. Those who had higher level of education in the study site (i.e., the owners) should have been more convinced by the As testing results. Thus, they came to place trust in this concrete fact, which overwhelmed their advanced knowledge on the risks of shallow tube-well water and, consequently, kept using the shallow tube-wells. In addition, the owners who relied more on the community tended to use shallow tube-wells. This tendency is understandable if we think how a belief is generally formed. The belief that the shallow tube-wells are safe should have been strengthened among the people tied strongly to each other. Thus, they kept using the shallow tube-wells for drinking purposes. As such, experiences shared in a community would affect the risk perceptions—this should be considered in risk communication programs [36].

In the cooking water source selection, determinants of water source choices worked differently for owners in the study site. Education and social capital were not associated with tap water use, but knowledge had a positive effect. A possible reason for this is that in the study site, the previous As testing result overwrote their risk perception so that the question might have measured another kind of attitude. In fact, if the question measured the degree of knowledge, the answer may have been related to educational level as it was for nonowners in the comparison site. The reason why more people tended to used tap water for cooking purposes than shallow tube-well water in both sites, particularly in the study site, was probably because of the fact that iron contents in nonfiltered groundwater from shallow tube-wells affected the color and taste of food, especially rice. Therefore, those from the study site who answered that they were more aware of the possibility of As contamination in shallow tube-well water were more likely to be concerned with the water quality, but they did not think of it as a matter of health.

Furthermore, as observed from the water source use and possession status in Table 4, people generally tended to choose the water sources that they owned for drinking purposes. The result coincides with the previous literature reporting the strong preference for own water sources in rural Bangladesh [37,38]. It can be inferred that the possession of a water source may limit people’s risk mitigation behavior. People in the areas studied may dislike asking neighbors to use a water source if they already have one and if they do not have an explicit reason to do so, such as the advantage of tap water use in cooking, particularly when neighbors are not relatives [24], or when one kind of social barrier exists in the region. On the other hand, nonowners do not have such a limitation. As a consequence, they might have been more likely to choose tap water than shallow tube-well.

### 5.3. Policy Implications

In the As-affected areas, underground water has a chance of being contaminated by As. In such areas, the groundwater quality should be frequently monitored when the water is provided as a drinking and cooking source. The pipeline system is ideal because the water supply is centrally controlled and monitored. Therefore, when the pipeline water is available, villagers should be mobilized to use tap water. However, as revealed from the analyses, when villagers have their own shallow tube-wells, they often use shallow tube-wells because it is less expensive and more convenient when the pipeline water supply is intermittent, and they tend to be bound to their own water sources. In such a situation, it may be not easy to change the attitudes of shallow tube-well owners. Naus et al. [30] also argued that it is generally challenging to get shallow tube-well users to switch their water source. They use shallow tube-wells out of habit and rely on availability, and thus, they are more attached to shallow tube-wells.

One viable option is to provide subsidies to enable villagers to obtain private taps. According to an estimate of the willingness to pay for piped water among villagers in the region [39], on average, the villagers were willing to pay BDT 960 for the initial capital cost of domestic connections and BDT 87 for the monthly recurring costs. These amounts are much less than the current cost levels: The minimum installation cost is about BDT 7000, and monthly tariffs are BDT 320 and BDT 100 in the study site and the comparison site, respectively. As the actual costs are much more expensive, especially for installation, people will not be inclined to install private taps and shift from using their owned shallow tube-wells. Another option is to install a public tap, which is economically more convenient for households. As was observed, owners are more likely to use their own water sources. However, if the water sources are provided as public goods, they may be more willing to use them. This option is also effective in securing safe water for the entire population, because the marginalized people often cannot afford to bear the cost for owning a private tap and they may not be allowed to use neighbors’ tap water. In India, rural water supplies provide taps to public properties. However, users’ unfavorable behaviors, such as returning back to tube-wells or constructing private taps illegally and connecting them to public water pipelines, have been reported [40]. Therefore, public tap installation itself may not change the situation essentially or it might cause other problems. Examining what combinations of public/private water sources and the pricing scheme effective to mobilize people is essential to secure safe water for all people and to prevent the rapid deterioration of underground water resources. The last, but certainly not least, option is to institutionalize the marketing and distribution of inexpensive and convenient water quality testing for households. This has not occurred. However, the initial national As testing was conducted with As field test kits, which cost just BDT 7,000 for 100 samples. Along with the fact that many newly installed, untested shallow tube-wells have caused around 50% of households to use untested water, this approach may just be the most effective option in the short term [6,41]. However, unless people are aware of the uncertainty in groundwater conditions, they may test their wells just once and assume one clean result is all that is necessary, as suggested by the results of the present study. This would not provide an accurate determination of water safety and would not be a desirable situation for arsenic testing distributors once this becomes a formalized business activity.

## 6. Conclusions

The study explored how the implementation actions have worked to mitigate the problem by looking at the situation of the village where the As was first reported in Bangladesh 25 years ago. A questionnaire survey and As tests on tube-well water were conducted in the village and in another neighboring village. The As levels in the tube-wells were found decreased in the study site. It is beyond the scope of this paper to examine the reason for this, but the case reported here seems worth investigating further, and hydrogeologic researchers are expected to extend the study. As for the villagers’ risk perception and behaviors, despite the enormous effort of the intervening bodies and the availability of safe water through pipelines in the two villages studied, information did not significantly induce the risk mitigation behaviors and a significant number of people continued to use shallow tube-wells. Furthermore, fewer respondents from the study site had sufficient knowledge about As compared to respondents from the comparison site, and they were more likely to use the nonfiltered shallow tube-wells although there have been many interventions in the study site since As contamination of the water was first reported 25 years ago. The possible reasoning for this tendency is that people are more likely to trust concerted evidence than general knowledge even though the evidence was provided occasionally. As such, the uncertainty about groundwater conditions is not well recognized by the general population. Therefore, practitioners should be careful when they share the As testing results with local people. Once they are told that their tube-wells are safe, they might believe the information for a long time. The As testing provides only tentative information about groundwater conditions. As economic conditions improve, people want to have their own water source. In most of the cases, shallow tube-wells are chosen and installed because they are economically viable and convenient, but they are not frequently monitored. Under such circumstances, considering people’s risk behavioral tendencies revealed through the analyses, certain measures are necessary to provide people with safe water. Providing subsides to install private taps, supplying public taps, and marketing and distributing handy water quality tests for households are suggested as viable options to mobilize people to get safe water. These measures will be more effective when they are combined with continuous enlightenment activities that further include the information on the nature of underground water.

## Figures and Tables

**Table 1:** Summary of previous scientific literature data.

Authors	Sampling Date(s)	Sample Size	Mean (µg/L)	Min (µg/L)	Max (µg/L)	% of Samples Greater than 50 µg/L	Figure 1 Legend
Khan et al. [2]	N/A	N/A	N/A	N/A	N/A	N/A	Blue line
Ahmad et al. [15] ^1^	N/A	25	601	10	2870	29.0%	Orange line
Ohno et al. [16]	April 2002	10	476	14	2630	70.0%	□
Reza et el. [17]	January 2008	20	50.6	2.76	170	40.0%	△
Reza et el. [18]	January 2008	20	62.4	3.02	315.15	35.0%	◇
Reza et el. [19]	N/A	20	50.6	2.76	170	40.0%	△
Reza et el. [20]	January 2008, 2009, 2010	54	48.81	2.76	315.15	N/A	N/A
Reza et el. [21]	January 2009	14	26.8	5.99	59.06	7.1%	☆
Islam et al. [22]	November/December 2014	18	66	3	151	57.9%	○

^1^ Among 25 randomly chosen samples. N/A represents that the information was not provided in the literature.

**Table 2:** As test results for the study site from the present and previous studies.

ID	Latitude	Longitude	As (µg/L) in 2018	As (µg/L) in 2014
Chamagram Village (the study site)				
1	24°36′15″	88°14′44″	21	
2	24°36′11″	88°14′41″	15	
3	24°36′15″	88°14′45″	28	
4	24°36′14″	88°14′45″	23	
5	24°36′13″	88°14′41″	12	
6	24°36′14″	88°14′43″	17	
7	24°36′11″	88°14′42″	16	
8	24°36′12″	88°14′45″	44	
9	24°36′08″	88°14′43″	39	
10	24°36′07″	88°14′42″	8	
Site from Islam et al. [22]				
1	24°36′12″	88°15′08″	3	32
2	24°36′16″	88°15′04″	3	101
3	24°36′16″	88°15′01″	1	40
4	24°36′15″	88°15′01″	11	N/A
5	24°36′14″	88°15′01″	9	110
6	24°36′15″	88°15′02″	3	80
7	24°36′17″	88°15′00″	2	25
8	24°36′16″	88°15′00″	5	N/A
9	24°36′19″	88°15′04″	5	N/A
10	24°36′20″	88°15′00″	7	61
11	24°36′20″	88°15′01″	2	46
12	24°36′20″	88°15′02″	2	25
13	24°36′18″	88°15′02″	2	151
14	24°36′14″	88°15′06″	1	20
15	24°36′11″	88°15′10″	12	145
16	24°36′14″	88°15′07″	2	61
17	24°36′13″	88°15′08″	1	N/A

N/A represents that these tube-wells were first tested in the present study and not tested in the previous study.

**Table 3:** Descriptive statistics of the questionnaire results.

Question	Total	Village	
		Study Site	Comparison Site
Literacy	149 (58.0)	73 (70.9)	76 (49.3)
Family monthly income (BDT) ^1^	13,490 (10,758)	15,233 (11,451)	12,340 (10,111)
Occupation			
Farmer (Farm owner)	17 (6.6)	1 (1.0)	16 (10.4)
Farm worker	18 (7.0)	3 (2.9)	15 (9.7)
Business owner	66 (25.6)	16 (15.5)	49 (31.8)
Business employee	17 (6.6)	7 (6.8)	10 (6.5)
Day laborer	90 (34.9)	40 (38.8)	50 (32.5)
Rickshaw puller/van driver	7 (2.7)	5 (4.9)	2 (1.3)
Remittance from other cities	11 (4.3)	11 (10.7)	0 (0.0)
Other	32 (12.4)	20 (19.4)	12 (7.8)
Own water source			
Yes	190 (73.9)	80 (77.7)	110 (71.4)
-Tap	24 (12.6)	6 (7.5)	18 (16.4)
-Shallow tube-well	156 (82.1)	66 (82.5)	90 (81.2)
-Tap & Shallow tube-well	10 (52.6)	8 (10.0)	2 (1.8)
Drinking water source			
Tap water	132 (51.2)	38 (36.9)	94 (61.0)
Nonfiltered shallow tube-well	123 (48.2)	64 (62.1)	59 (38.3)
Filtered shallow tube-well	2 (0.8)	1 (1.0)	1 (0.6)
Cooking water source			
Tap water	168 (65.4)	71 (68.9)	97 (63.0)
Nonfiltered shallow tube-well	88 (34.2)	32 (31.1)	56 (36.4)
River water	1 (0.4)	0 (1.0)	1 (0.6)
Bathing water source			
Tap water	142 (55.3)	50 (48.5)	92 (59.7)
Nonfiltered shallow tube-well	114 (44.4)	53 (51.5)	61 (39.6)
Pond water	1 (0.4)	0 (0.0)	1 (0.6)
Arsenicosis cases			
In family (Yes)	15 (5.8)	0 (0.0)	15 (9.7)
-Number of patients ^1,2,3^	2.68 (4.56)	0 (0.0)	2.68 (4.56)
-Duration of illness (in years) ^1,2,3^	10.5 (10.3)	0 (0.0)	10.5 (10.3)
In neighborhood (Yes)	30 (11.7)	0 (0.0)	30 (19.5)
Number of respondents	257 (100.0)	103 (40.1)	154 (59.9)

^1^ Figures outside of parentheses indicate the number of applicable respondents and figures inside parentheses represent the percentage of the column totals except in the case of family monthly income, number of patients, and duration of illness. For these, the figures outside of parentheses show the mean, and figures inside parentheses represent standard deviations. ^2^ per household with arsenicosis patient. ^3^ based on the answers reported by respondents.

**Table 4:** Comparison of water source uses and possession status between the two villages.

Use	Total	Owners				Nonowners
		Tap	Shallow Tube-Well	Both	Total	
Drinking Water Study site						
Tap	38 (36.9)	5 (83.3)	14 (21.2)	3 (37.5)	22 (27.5)	16 (69.6)
Shallow tube-well	65 (63.1)	1 (16.7)	52 (78.8)	5 (62.5)	58 (72.5)	7 (30.4)
Total	103 (100)	6 (100)	66 (100)	8 (100)	80 (100)	23 (100)
Comparison site						
Tap	94 (61.0)	16 (88.9)	37 (41.1)	2 (100)	55 (50.0)	39 (88.6)
Shallow tube-well	60 (39.0)	2 (11.1)	53 (58.8)	0 (0)	55 (50.0)	5 (11.4)
Total	154 (100)	18 (100)	90 (100)	2 (100)	110 (100)	44 (100)
Cooking Water Study site						
Tap	71 (68.9)	5 (83.3)	41 (62.1)	6 (75.0)	52 (65.0)	19 (82.6)
Shallow tube-well	32 (31.1)	1 (16.7)	25 (37.9)	2 (25.0)	28 (35.0)	4 (17.4)
Total	103 (100)	6 (100)	66 (100)	8 (100)	80 (100)	23 (100)
Comparison site						
Tap	97 (63.0)	16 (88.9)	40 (44.4)	2 (100)	58 (50.0)	39 (88.6)
Shallow tube-well	56 (36.4)	2 (11.1)	50 (55.6)	0 (0)	52 (50.0)	4 (9.1)
River	1 (0.6)	0 (0)	0 (0.0)	0 (0)	0 (0)	1 (2.3)
Total	154 (100)	18 (100)	90 (100)	2 (100)	110 (100)	44 (100)
Bathing Water Study site						
Tap	50 (48.5)	5 (83.3)	21 (31.8)	6 (75.0)	32 (40.0)	18 (78.3)
Shallow tube-well	53 (51.5)	1 (16.7)	45 (68.2)	2 (25.0)	48 (60.0)	5 (21.7)
Total	103 (100)	6 (100)	66 (100)	8 (100)	80 (100)	23 (100)
Comparison site						
Tap	92 (59.7)	16 (88.9)	35 (38.9)	2 (100)	53 (48.2)	39 (88.6)
Shallow tube-well	61 (39.6)	2 (11.1)	54 (60.0)	0 (0)	56 (50.9)	5 (11.4)
River	1 (0.6)	0 (0)	1 (1.1)	0 (0)	1 (0.9)	0 (0.0)
Total	154 (100)	18 (100)	90 (100)	2 (100)	110 (100)	44 (100)

Figures outside of parentheses show counts of applicable respondents and figures inside parentheses represent the percentage of the column totals for each village.

**Table 5:** Logistic regression results for determining likelihood of tap water installation (N = 257) ^1^.

	Dependent Variable: Tap Installation (0 = Not Possessing a Tap, 1 = Possessing a Tap)			
Explanatory Variables	(1)	(2)	(3)	(4)
Log of income	0.272	0.298	0.274	0.296
	(0.249)	(0.267)	(0.250)	(0.264)
Education ^2^	0.143		0.144	0.144
	(0.092)		(0.092)	(0.092)
Feeling physical burden ^3^	−0.032	−0.033	−0.032	−0.036
	(0.186)	(0.186)	(0.186)	(0.187)
Knowledge on shallow tube-well ^4^	−0.100	−0.118		−0.139
(Knowledge)	(0.258)	(0.256)		(0.262)
Social capital ^5^	0.117	0.100	0.117	
	(0.246)	(0.250)	(0.247)	
Village	0.012			
(0 = Study site, 1 = Comparison site)	(0.390)			
Village × Education				
—Study site		0.099		
		(0.114)		
—Comparison site		0.167		
		(0.097)		
Village × Knowledge				
—Study site			−0.106	
			(0.265)	
—Comparison site			−0.096	
			(0.253)	
Village × Social capital				
—Study site				0.077
				(0.256)
—Comparison site				0.150
				(0.252)
Intercept	−4.880	−4.976	−4.889	−4.963
	(2.990)	(3.129)	(2.992)	(3.108)
McFadden’s pseudo r-squared	0.022	0.025	0.022	0.025
AIC	210.4	200.90	210.39	209.91

^1^ Figures outside of parentheses show the estimated coefficients and figures inside parentheses represent standard errors. ^2^ 7 raking (1 = no experience, 2 = primary drop out, 3 = primary, 4 = high school dropout, 5 = high school, 6 = college, 7 = university). ^3^ 5-point Likert scale (1 = Strongly no, 5 = Strongly yes). ^4^ 5-point Likert scale (1 = Don’t know, 5 = Well know) ^5^ 5-point Likert scale answer to question “How much ratio of people do you have high reliability on in this village?” (1 = Hardly, 2 = Less than half, 3 = About half, 4 = More than half, 5 = Almost completely).

**Table 6:** Logistic regression results for determining likelihood of drinking water source selection (N = 255) ^1^.

	Dependent Variable: Water Source (0 = Shallow Tube-Well, 1 = Tap)				
Explanatory Variables	(1)	(2)	(3)	(4)	(5)
Log of income	0.021	0.074	0.026	0.062	0.085
	(0.114)	(0.124)	(0.121)	(0.125)	(0.131)
Education	−0.015	0.004		0.032	0.023
	(0.080)	(0.079)		(0.082)	(0.083)
Feeling physical burden	−1.415 ***	−1.185 ***	−1.367 ***	−1.271 ***	−1.325 ***
	(0.180)	(0.172)	(0.183)	(0.184)	(0.192)
Knowledge	0.152	0.306	0.292		0.100
(Knowledge)	(0.226)	(0.220)	(0.228)		(0.233)
Social capital	−0.396	−0.222	−0.405	−0.354	
	(0.213)	(0.203)	(0.229)	(0.216)	
Village	1.392 ***				
(0 = Study site, 1 = Comparison site)	(0.346)				
Possession (0 = No, 1 = Yes) ^2^		−1.569 ***			
		(0.416)			
Village × Possession × Education					
—Nonowners in the study site			−0.104		
			(0.160)		
—Owners in the study site			−0.300 **		
			(0.111)		
—Nonowners in the comparison site			0.529 *		
			(0.233)		
—Owners in the comparison site			0.088		
			(0.088)		
Village × Possession × Knowledge					
—Nonowners in the study site				0.349	
				(0.275)	
—Owners in the study site				−0.008	
				(0.234)	
—Nonowners in the comparison site				0.824 **	
				(0.276)	
—Owners in the comparison site				0.323	
				(0.226)	
Village × Possession × Social capital					
—Nonowners in the study site					−0.127
					(0.261)
—Owners in the study site					−0.633 **
					(0.232)
—Nonowners in the comparison site					0.264
					(0.267)
—Owners in the comparison site					−0.221
					(0.210)
Intercept	4.236	3.888	4.441	3.487	3.855
	(1.764)	(1.819)	(1.895)	(1.882)	(1.953)
McFadden’s pseudo r-squared	0.298	0.294	0.330	0.336	0.356
AIC	261.33	263.2	254.75	252.44	245.43

*** *p* < 0.001, ** *p* < 0.01, * *p* < 0.05. ^1^ The two respondents whose answer to the question about the drinking water source was “Filtered shallow tube-well” were excluded from the whole sample of 257. ^2^ Possession of any kind of water source.

**Table 7:** Logistic regression results for determining likelihood of cooking water source selection (N = 256) ^1^.

	Dependent Variable: Water Source (0 = Shallow Tube-Well, 1 = Tap)				
Explanatory Variables	(1)	(2)	(3)	(4)	(5)
Log of income	0.017	0.022	0.019	0.020	0.031
	(0.102)	(0.105)	(0.104)	(0.105)	(0.106)
Education	0.066	0.084		0.105	0.103
	(0.076)	(0.077)		(0.079)	(0.078)
Feeling physical burden	−0.992 ***	−0.873 ***	−0.935 ***	−0.904 ***	−0.882 ***
	(0.164)	(0.168)	(0.165)	(0.172)	(0.171)
Knowledge on shallow tube-well	0.427 *	0.366	0.413		0.416 *
(Knowledge)	(0.210)	(0.204)	(0.206)		(0.210)
Social capital	0.034	−0.067	0.102	0.112	
	(0.188)	(0.191)	(0.193)	(0.193)	
Village	−0.504				
(0 = Study site, 1 = Comparison site)	(0.315)				
Possession (0 = No, 1 = Yes)		−1.232 **			
		(0.438)			
Village × Possession × Education					
—Nonowners in the study site			0.217		
			(0.155)		
—Owners in the study site			0.115		
			(0.100)		
—Nonowners in the comparison site			0.424 *		
			(0.206)		
—Owners in the comparison site			0.013		
			(0.082)		
Village × Possession × Knowledge					
—Nonowners in the study site				0.489	
				(0.271)	
—Owners in the study site				0.481 *	
				(0.219)	
—Nonowners in the comparison site				0.734 **	
				(0.254)	
—Owners in the comparison site				0.239	
				(0.207)	
Village × Possession × Social capital					
—Nonowners in the study site					0.269
					(0.257)
—Owners in the study site					0.130
					(0.198)
—Nonowners in the comparison site					0.420
					(0.250)
—Owners in the comparison site					−0.060
					(0.190)
Intercept	2.038	2.360	1.225	1.153	1.022
	(1.606)	(1.626)	(1.643)	(1.648)	(1.647)
McFadden’s pseudo r-squared	0.173	0.192	0.186	0.213	0.208
AIC	286.56	280.12	286.18	277.84	279

*** *p* < 0.001, ** *p* < 0.01, * *p* < 0.05. ^1^ The one respondent whose answer to the question about the cooking water source was “River” was excluded from the whole sample of 257.

## Data Availability

The data presented in this study are available on request from the corresponding author. The data are not publicly available due to ethical restrictions.
